# Study on the transferability of the knowledge-based VMAT model to predict IMRT plans in prostate cancer radiotherapy

**DOI:** 10.1186/s40001-023-01278-1

**Published:** 2023-08-31

**Authors:** Suyan Bi, Xingru Sun, Wan Fatihah Binti Wan Sohaimi, Ahmad Lutfi Bin Yusoff

**Affiliations:** 1 Department of Nuclear Medicine, Radiotherapy & Oncology, School of Medical Sciences, Universiti Sains Malaysia, Health Campus, 16150, Kubang Kerian, Kelantan Malaysia; 2 Department of Radiation Oncology, National Cancer Center/National Clinical Research Center for Cancer/Cancer Hospital & Shenzhen Hospital, Chinese Academy of Medical Sciences and Peking Union Medical College, Shenzhen, 518116 Guangdong China; 3 Hospital Universiti Sains Malaysia, Health Campus, 16150, Kubang Kerian, Kelantan Malaysia

**Keywords:** Knowledge-based planning, VMAT automated planning model, Model transfer, Prostate radiotherapy, IMRT planning

## Abstract

**Objective:**

The aim of this study was to investigate the feasibility of VMAT library-derived model transfer in the prediction of IMRT plans by dosimetry comparison among with three groups of IMRT plans: two groups of automatic IMRT plans generated by the knowledge-based the volumetric modulated arc therapy (VMAT) model and intensity-modulated radiation therapy (IMRT) model and one group of manual IMRT plans.

**Methods:**

52 prostate cancer patients who had completed radiotherapy were selected and randomly divided into 2 groups with 40 and 12 separately. Then both VMAT and IMRT plans were manually designed for all patients. The total plans in the group with 40 cases as training datasets were added to the knowledge-based planning (KBP) models for learning and finally obtained VMAT and IMRT training models. Another 12 cases were selected as the validation group to be used to generated auto IMRT plans by KBP VMAT and IMRT models. At last, the radiotherapy plans from three groups were obtained: the automated IMRT plan (V-IMRT) predicted by the VMAT model, the automated IMRT plan (I-IMRT) predicted by the IMRT model and the manual IMRT plan (M-IMRT) designed before. The dosimetric parameters of planning target volume (PTV) and organ at risks (OARs) as well as the time parameters (monitor unit, MU) were statistically analyzed.

**Results:**

The dose limit of all plans in the training datasets met the clinical requirements. Compared with the training plans added to VMAT model, the dosimetry parameters have no statistical differences in PTV (*P* > 0.05); the dose of X% volume (Dx%) with D25% and D35% in rectal and the maximum dose (Dmax) in the right femoral head were lower (*P* = 0.04, *P* = 0.01, *P* = 0.00) while D50% in rectal was higher (< 0.05) in the IMRT model plans. In the 12 validation cases, both automated plans showed better dose distribution compared with the M-IMRT plan: the Dmax of PTV in the I-IMRT plans and the dose in volume of interesting (VOI) of bladder and bilateral femoral heads were lower with a statistically significant difference (P < 0.05). Compared with the I-IMRT plans, dosimetric parameters in PTV and VOI of all OARs had no statistically significant differences (*P* > 0.05), but the Dmax in left femoral heard and D15% in the right femoral head were lower and have significant differences (*P* < 0.05). Furthermore, the low-dose regions, which was defined as all volumes outside of the PTV (RV) with the statistical parameters of mean dose (Dmean), the volume of covering more than 5 Gy dose (V5Gy), and also the time parameter (MU) required to perform the plan were considered. The results showed that Dmean in V-IMRT was smaller than that in the I-IMRT plan (*P* = 0.02) and there was no significant difference in V5Gy and MU (*P* > 0.05).

**Conclusion:**

Compared with the manual plan, the IMRT plans generated by the KBP models had a significant advantage in dose control of both OARs and PTV. Compared to the I-IMRT plans, the V-IMRT plans was not only without significant disadvantages, but it also achieved slightly better control of the low-dose region, which meet the clinical requirements and can used in the clinical treatment. This study demonstrates that it is feasible to transfer the KBP VMAT model in the prediction of IMRT plans.

## Introduction

Modern radiation therapy employs intensity modulation, which uses the inverse optimization technique to produce the corresponding dose distribution from the optimum target conditions based on the clinical target and organs at risk (OARs) dose objectives. Individual experiences and the amount of effort spent developing a plan impact a plan’s quality to a great extent [1], as different dose objectives lead to different optimization results. Manual planning has two drawbacks. First, there are no universal and unbiased quality control standards for the plans, and environmental, psychological, and personal experience elements all affect the quality and stability of the plans [2–4]. Second, the process of developing a plan is labor and time intensive since it requires a lot of mechanical and repetitive effort, such as structure processing, condition setup, and iterative plan optimization [5]. For these reasons, using machine learning to construct radiotherapy plans has emerged as a popular area of study [6]. Knowledge-based planning (KBP) is a machine learning strategy that creates the best possible treatment plan for a disease by incorporating empirical parameters from earlier plans into a new plan optimization [7, 8]. Numerous institutions both domestically and abroad have used KBP to create different intensity-modulated regimens for prostate, lung, and head and neck malignancies, and so on [9, 10]. The results of various research have demonstrated that the quality of predicted plans can be on par with or better than that of manual plans, and that they are also significantly more stable [3, 5, 11]. In 2011, Kevin L. Moore et al. [3] first proposed that factors such as the relationship in the dose objectives between the target region and OAR, and the variations in geometric location can be used as quantitative indicators of the plan quality [4, 12]. Based on statistical discrepancies, Batumalai V [5] observed that even the engagement of experienced plan developers does not necessarily guarantee the variations in plan quality. Wu Hao et al. [13] also concluded that intensity-modulated planning using KBP, along with manual fine-tuning, was not only able to achieve a clinically acceptable level in almost all of the plans but also significantly reduced the planning time. This was based on a comparison of automated IMRT plans with conventional manual plans in 80 patients with cervical cancer. RapidPlan^™^ automated planning model (TPS, Varian Medical Systems, Version 13.6, Inc.), which based on the automatic optimization of DVH, is one of automated modeling methods built on KBP. The training database is used to extract the characteristics of the training structures, and the geometry-based expected dose (GED) technique is used to produce the anticipated dose volume histogram (DVH) for PTV and OAR. The OARs are then split into four groups based on their positions about the target region: the target-overlap region, the in-field region, the leaf-transmission region, and the out-of-field region [14, 15]. The in-field region, which has the largest OAR dosage changes in the anticipated DVH, is the area on which the principal component analysis is conducted. By integrating additional influencing conditions, the primary influencing elements of the specific structure’s dose distribution are then identified. The test design is continuously optimized using the model until it satisfies the clinical requirements. As the automated planning increasing in popularity, many issues regarding the application of models deserve consideration [13]. Based on a review of the literature, we discovered that different radiotherapy centers frequently use various irradiation techniques depending on the actual situation when developing plans for the same disease, whereas the currently established KBP models were derived from a database of the same techniques and are only used to predict plans for the same techniques [16]. In practice, it takes a long time to model various procedures for the same site, and it is difficult for radiotherapy clinics with rare number patients to collect the case. As a result, this study used the volumetric modulated arc therapy (VMAT) and the intensity-modulated radiation therapy (IMRT) models of the KBP to separately predict IMRT plans, in order to determine the feasibility of using the VMAT model to predict IMRT plans and investigate the usability of model transfer.

## Materials and method

**Fig. 1:**
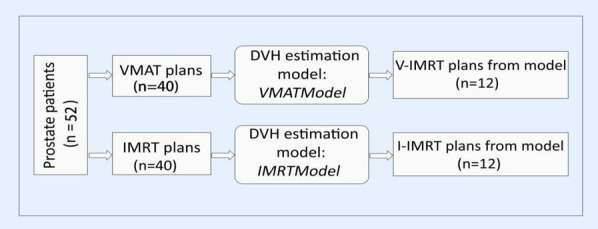
Flowchart of the research protocol

The case selection, plan development, model training, and results prediction were four primary components of the experimental protocol design. The experimental protocol’s flowchart is shown in Fig. 1.

### Case selection and manual plan development

The 52 patients who only underwent external radiation treatment for prostate cancer were chosen [17]. Their age was range from 43 to 75, with a median of 58 years old, and TNM stages of T2b to T3. Supine position was employed for CT positioning. The scanning thickness was 3 mm, the scanning was range from the lower edge of the lumbar 4 to 3 cm below the ischial tuberosity, and the fixing method used a thermoplastic mask. The delineation of the clinical target volume (CTV) included the entire prostate gland, the seminal vesicle gland area, and the pelvic lymphatic drainage. The external iliac lymph nodes, internal iliac lymph nodes, obturator lymph nodes, and some of the common iliac lymph nodes were all part of the pelvic lymphatic drainage area. The PTV was created by expanding the CTV in the anterior–posterior, left-to-right, and superior–inferior directions, respectively, by 0.8–1 cm, 0.7–0.8 cm, and 0.5–1 cm. The rectum, bladder, left and right femoral heads, as well as small intestine were included in the OARs delineation [18, 19]. VMAT and IMRT plans of each case were created separately. The IMRT plan was given 7 fields with equal angle between 0–360° and the X-axis of the collimator paralleling to the longest diameter in each field from the beam's eye view (BEV). The VMAT was given two complete arcs with a 5–10° rotation of the collimator. The analytical anisotropic algorithm (AAA) was chosen as the optimization algorithm for both plans, with the prescribed dose in the primary lesion area was 69.3 Gy/30 doses. 6MV X-rays were employed for both plans. The target region requires the prescribed dose to cover more than 95% of the volume and the dose limitation of the OARs were as follow: with a maximum dose of 71 Gy for the rectum and bladder, less than 5% volumes of the right and left femoral head receiving a dose of 50 Gy, the maximum dose for the small intestine is 71 Gy but its volume that receives 15 Gy dose were kept below 120 cc. The doses of other volumes not specified in the dose objectives were kept as low as possible. After the above plans were completed by the Eclipse planning system (TPS, Varian Medical Systems, Version 13.6, Inc.), they were then conducted plan quality assessment by senior physicians.

### KBP modeling

A training dataset (40 cases) and a test dataset (12 cases) were created by randomly dividing the aforementioned examples into two groups. RapidPlanTM was used to generate the VMAT and IMRT models for the KBP. Here, the optimization conditions for each structure involved in the training were set. The model was built using the following procedures:

 Creation of a new model and improvement of the pertinent data: the primary lesion, rectum, bladder, left, and right femoral heads were chosen as the training target structures. At least one target region had to be included in the training structures. The lymphatic drainage area and the small intestine were not included in the training model in this study because the treatment range of the chosen cases only included the primary lesion while in some cases, the clinical delineation placed them far from the primary lesion. The dose of the small intestine was evaluated in the dosimetric statistics only for reference.

 Model training and eigenvalue extraction: The appropriate plan from the training sample was chosen to be incorporated into the model, parameters of structure were extracted, then the model training was carried out. To create each planned Geometric DVH (GEDVH) in accordance with the correlation between the radiation field and the target geometric point, the model training was divided into two parts. The average DVH and the principal components of the DVH of each target structure were then determined by performing the principal component analysis on the DVH and GEDVH of all plans. The DVH of each target structure was then predicted using a mix of geometric parameters, including position and shape, using a regression model that was constructed based on the determined principal components.

 Statistical analysis and model cleaning: Following model training, the model was assessed and improved in accordance with the statistical findings. First, the goodness-of-fit coefficient determination *R*^2^ was inspected. *R*^2^ was then adjusted to be between 0.7 and 0.9 by modifying the training plans [20]. The DVH plots of each PTV were then examined. The DVHs of the models participating in the training plans needed to be relatively more concentrated, and separate analysis was required for plans with relatively greater disparities in DVH distribution. The residual plots of each training structure were then examined; it was necessary to examine each of the anomalous discrete points separately. To determine whether the reference values of the structures in the plan surpassed the threshold, one might examine the summary plot, a red color is shown whenever a threshold is exceeded. Before including a plan in the model, it had to be evaluated for removal or re-optimization if two or more reference values in the plan exceeded the threshold [21]. Reference values for the man- agement and summary plot: (a) Cook’s Distance (CD): The cutoff value is 10.0, and a CD of greater than 10 indicates that the plan includes dose anomalies that should be the assessment’s top priority. (b) Studentized residual values (SR): These statistical outliers have a threshold of 3.0, and their results are a direct reflection of how closely the data adhere to the fitted curve. In general, the plan points that are anomalous in the regression or residual plots have a considerably higher SR value. (c) Modified Z-score (MZ). The threshold for this number, which assesses the geometric relationship between OAR and PTV, is 3.5. If the structure is not discovered to have any delineation mistakes, the deletion of plans is typically not carried out in accordance with the MZ values due to the MZ values’ relatively higher variability [22]. (d) A real difference of estimate (dA): This number could be abnormal if the volume variation is comparable to the MZ value or relatively larger. It is generally not used as a criterion for plan deletion.

 Setting the intended optimization objective for the finished training structure. The optimization target may be defined using individually made planning objectives or with limits that are automatically created by the model. In most cases, it is required to manually increase the conditional limitations when the automatically created limits are insufficient to satisfy the plan’s needs.

### Model clinical validation and verification

Clinical testing of the VMAT model and IMRT model plans acquired from the training was done using 5 randomly chosen patients from the training dataset. All settings conditions of the manual plan were maintained, and the training model was added to the plan optimization interface for automatic optimization. The model was made available by adjusting the model target optimization parameters until the DVH dose lines of all OARs fell within the shaded area of the automated optimization. Following the conclusion of model testing, the remaining 12 cases served as a validation dataset. IMRT automated planning was then performed using the VMAT model and IMRT model individually, resulting in the V-IMRT, I-IMRT, and the previously finished M- IMRT planning groups. The plans of the three groups’ PTV and OARs dosimetric parameters were compared. The target region dose homogeneity index (HI) and conformity index (CI), D15%, D25%, D35%, and D50% of the bladder and rectum, as well as Dmax, D40%, and D25% of the right and left femoral heads, were among them. The V-IMRT and I-IMRT plans’ mean doses (Dmean) for the PTV and areas outside of it (RV) were tallied, and the monitor unit (MU) used during the treatment period was assessed.

### Statistical analysis

Statistics were expressed as x ± s. Paired samples t-test was performed using SPSS 22.0. A *P* < 0.05 was considered statistically significant.

## Results

### Dosimetry comparison of the plans of the two groups in the training dataset

Following radiation oncologists’ approval, all plans may be employed in clinical treatment because they all complied with prescription standards. The statistical results of dose distribution from PTV and OARs for the two groups of plans are shown in Table1. The VMAT plan group demonstrated slightly better control of the hot spots in PTV than the IMRT plan group, while no appreciable variations in HI and CI in the target region were observed. The control of rectal dose was a key factor in the comparison of OARs. Here, IMRT had a benefit for controlling the volumetric dose D25 percent (*P* = 0.040). VMAT, however, outperformed IMRT for the management of both volumetric doses, D35% and D50% (*P* = 0.01, *P* = 0.040). The VMAT plan was also significantly superior to IMRT for restricting the maximal dosage to the right femoral head (*P* = 0.000).

**Table 1:** Dosimetric statistics of PTV and OAR for the two plans in the training dataset

	Plan added to the IMRT model	Plan added to the VMAT model	*P* value
PTV			
Dmax (Gy)	73.21 ± 0.61	72.73 ± 1.29	0.051
V105% (cc)	0.11 ± 0.30	0.18 ± 0.43	0.38
HI	1.06 ± 0.01	1.07 ± 0.02	0.11
CI	0.89 ± 0.03	0.89 ± 0.03	0.13
Rectum (Gy)			
D15%	52.86 ± 7.47	52.53 ± 6.59	0.89
D25%	40.13 ± 7.01	42.46 ± 7.11	0.04
D35%	31.73 ± 7.14	35.02 ± 6.65	0.01
D50%	21.10 ± 6.75	15.97 ± 15.5	0.04
Bladder(Gy)			
D15%	35.30 ± 17.24	36.32 ± 18.84	0.22
D25%	25.09 ± 17.44	25.19 ± 19.99	0.91
D35%	20.32 ± 16.1	20.23 ± 18.11	0.93
D50%	14.90 ± 12.85	15.97 ± 15.5	0.26
Lt femur (Gy)			
Dmax	29.25 ± 7.10	36.05 ± 6.73	3.27
D40%	14.41 ± 4.29	14.38 ± 7.60	0.98
D25%	17.12 ± 5.18	19.56 ± 8.54	0.09
Rt femur (Gy)			
Dmax	29.15 ± 7.05	34.83 ± 6.95	0.00
D40%	15.65 ± 4.47	14.96 ± 7.09	0.57
D25%	18.87 ± 5.99	19.87 ± 7.83	0.49

### Outlier management and statistical validation results

**Fig. 2:**
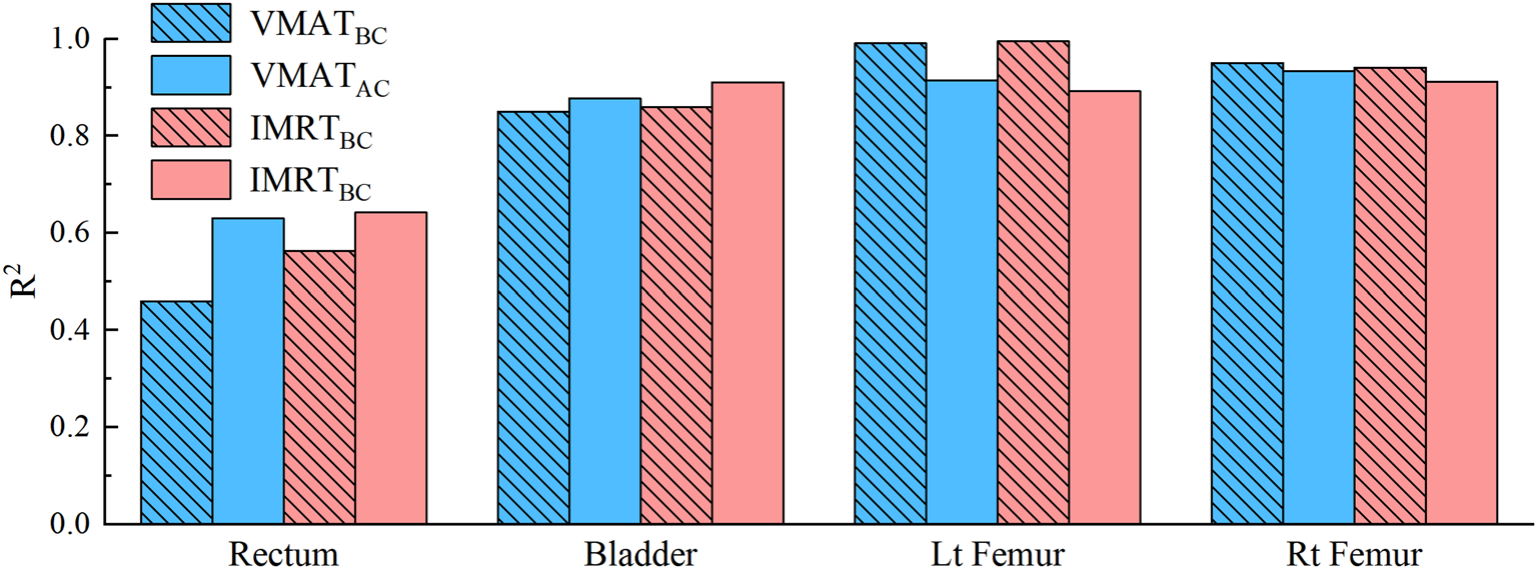
Changes in *R*^2^ values before and after the cleaning of the model

During the statistical validation of the two models, the training plans corresponding to the substantially larger outliers in the VMAT model and the IMRT model (7 cases for the VMAT model and 8 cases for the IMRT model) were discarded. The VMAT model eventually had 33 plans, and the IMRT model had 32 plans. In 2 cases, the initial designs were amended. Figure 2 shows the comparison of *R*^2^ values from various target before and after the two models were cleaned.

**Fig. 3:**
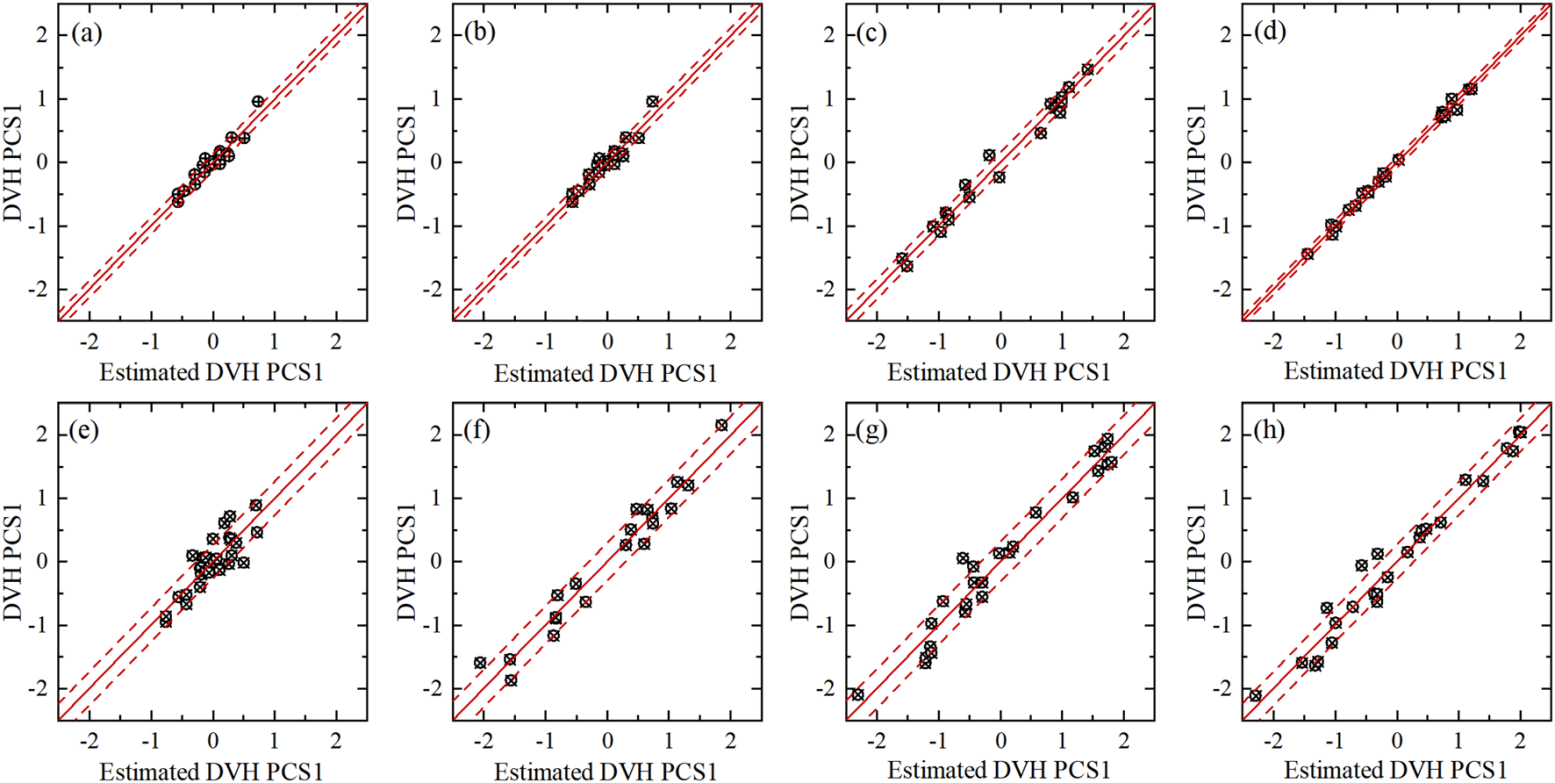
Residual plots between the actual results and the predicted results of DVH principal component analysis of OARs. In the IMRT model, the rectum, bladder, left femoral head, and right femoral head are, respectively, (**a**) through (**d**), and in the VMAT model group, they are, respectively, (**e**) through (**h**). The first primary component score (PCS1) of the predicted DVH for each plan’s structure is represented by the horizontal coordinate, and the PCS1 of the corresponding actual DVH of the structures is represented by the vertical coordinate. The figure’s dashed line denotes the error range, while the solid line shows that the predicted and actual numbers are equivalent

**Fig. 4:**
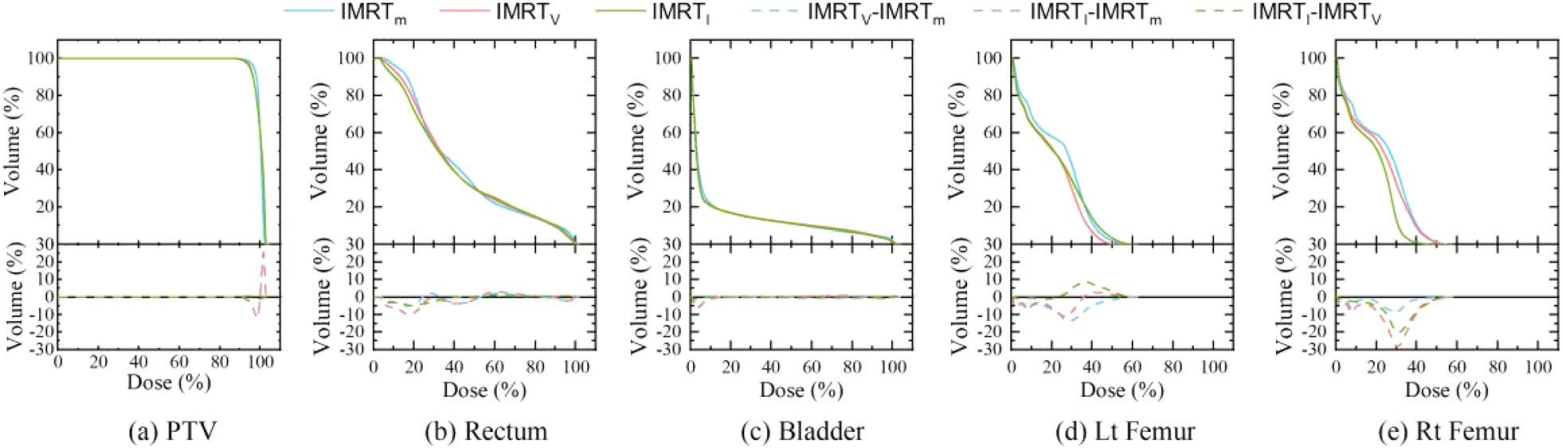
Comparison of DVH of PTV (**a**) and OAR[rectum (**b**), bladder (**c**), Lt femur (**d**) and Rt femur (**e**)] of IMRT plan generated by two models with manual IMRT plan

Figure 2 demonstrates that following cleaning, the *R*^2^ of the rectum, bladder, and left femoral head all improved, with the rectum of the VMAT model showing the greatest improvement. The *R*^2^ values of right femoral head, nevertheless, were both relatively good before and after the cleaning. The residual plots of the model after cleaning are shown in Figure 3 and are used to assess how well the regression equations from the principal component analysis fit into the model. The first primary component score (PCS1) of the predicted DVH for each plan’s structure is represented by the horizontal coordinate, and the PCS1 of the corresponding actual DVH of the structures is represented by the vertical coordinate. The figure’s dashed line denotes the error range, while the solid line shows that the predicted and actual numbers are equivalent. Figure 4 shows that neither of the models departed from the larger outliers. The fitting accuracy of the IMRT model developed in this work was somewhat higher than that of the VMAT model, while the residual values of every structure were all lower and the overall dispersion was smaller in the IMRT model.

### Dosimetric comparison of the target region and OAR in the validation dataset

Table 1 contains the findings from the comparison of the dose distributions of PTV and OARs for the M-IMRT, V-IMRT, and I-IMRT plans. In all cases, the dose distributions in the target regions complied with the clinical requirements. As evident from the above table, there were no statistical differences between the three groups, which means that the disparities between the the PTV Dmax and V105% among the plans of the three groups were not significant, with the IMRT model group having the smallest difference. Apart from the statistically significant difference between the HI values of the manual plan and IMRT group plan (*P* = 0.04), there were no statistically significant difference between the HI and CI values of the PTV between the manual plan and those of the model groups (*P* > 0.05).

The statistics of OARs between the two model group plans did not have significantly difference, as shown in Table 2. But the two model plans have a better dose distribution than the M-IMRT plans which receiving lower dosage to the bladder and bilateral femoral heads as compared to M-IMRT (*P* < 0.05). The DVH plots of the PTV, rectum, bladder, and bilateral femoral heads of the plans in three groups are shown in Fig. 4(a) through (e), where solid, dashed, and dotted lines are employed, respectively, for the IMRT model group, VMAT model group, and manual group. There was minimal difference in the high-dose region of the OARs between the dose control of all the structures in the model group plans and that of the manual plans. However, the dose differences between the femoral head and the low-dose region of the bladder were clear.

**Table 2:** Comparisons of the PTV and OAR doses between the manual plan and the two IMR automated plans (*x*¯ ± *s*)

	M-IMRT(M)	V-IMRT(V)	I-IMRT(I)	*P* value		
				M&V	V&I	M&I
PTV						
Dmax (Gy)	73.95 ± 1.04	73.22 ± 0.87	72.90 ± 0.59	0.54	0.65	0.89
V105% (cc)	0.12 ± 0.25	0.01 ± 0.01	0.00 ± 0.01	0.17	0.21	0.88
HI	1.05 ± 0.02	1.04 ± 0.01	1.04 ± 0.01	0.12	0.04	0.57
CI	0.87 ± 0.03	0.89 ± 0.01	0.86 ± 0.02	0.64	0.61	0.58
Rectum(Gy)						
D15%	51.68 ± 2.69	52.98 ± 5.53	52.51 ± 5.31	0.25	0.51	0.65
D25%	41.85 ± 2.57	42.85 ± 6.19	41.92 ± 6.37	0.60	0.97	0.46
D35%	34.88 ± 2.12	34.84 ± 5.94	33.69 ± 7.10	0.99	0.65	0.48
D50%	25.63 ± 2.77	23.45 ± 5.67	23.92 ± 7.36	0.35	0.55	0.66
Bladder(Gy)						
D15%	45.36 ± 13.85	38.84 ± 17.74	39.85 ± 17.55	0.20	0.28	0.06
D25%	37.27 ± 17.54	25.88 ± 18.45	26.54 ± 18.45	0.05	0.07	0.01
D35%	32.01 ± 16.33	17.69 ± 16.36	17.77 ± 16.36	0.01	0.01	0.88
D50%	25.94 ± 15.49	10.54 ± 12.42	10.36 ± 12.58	0.01	0.01	0.67
Left femoral head(Gy)						
Dmax	40.24 ± 6.90	35.28 ± 7.48	35.13 ± 6.65	0.02	0.01	0.88
D40%	20.17 ± 8.28	15.99 ± 7.36	16.44 ± 7.53	0.01	0.01	0.04
D25%	24.22 ± 9.14	19.25 ± 8.37	19.59 ± 8.23	0.01	0.01	0.35
Right femoral head(Gy)						
Dmax	37.92 ± 11.57	28.74 ± 11.57	31 ± 9.72	0.00	0.01	0.04
D40%	19.68 ± 8.72	12.67 ± 7.2	13.42 ± 6.87	0.00	0.01	0.24
D25%	24.11 ± 9.12	16.71 ± 8.13	17.6 ± 8.11	0.00	0.01	0.20

## Discussion

The application principle based on KBP is to integrate previously obtained plan development experiences into the intensity-modulated radiation optimization process to obtain the best possible treatment plan according to the previously published model [23]. Before the models can be used clinically, they must firstly be established, statistically evaluated, iteratively cleansed, and validated using clinical plans. Both models developed in current work can satisfy clinical requirements.

The *R*^2^ value, as the final evaluation criterion for the model training, should be controlled within specific range. When the *R*^2^ value is too small, the model structure is underfitting and cannot provide enough prediction details, which results in a poor prediction effect. When the *R*^2^ value is too large, the model structure is overfitting and may provide too many details, which weakens the model’s ability to generalize [23, 24]. The value should be controlled within the required range. All models with deleted plans must go through training again until all values are within the normal range and optimized for the given conditions.

It is clear from results in Table 2 and Figure 4, dosimetric analysis results of PTV and OARs, DVH analysis results for all training structures, which the quality of the automated plans obtained in the test database were on par with or better than the manual plans. The dose distributions of the OARs in the V-IMRT and the I-IMRT plans were similar when comparing the plans predicted by the two model groups. In order to exclude the influence of dose distribution of training plan sets in two models on the quality of the predicted model, the doses of VOI from PTV and OARs were recorded and analyzed as well, which are indicated in Table 1. The results showed that the data from the training plans of IMRT and VMAT model did not significantly difference on the dose distribution of PTV and OARs. In addition, the training plans in VMAT model demonstrated a little inferior fitting accuracy compared to the plans in IMRT model when the dispersion of the model was statistically examined. For example, the right femoral head had a higher dosage in the training plans of VMAT model than that in the IMRT model. Even though the following results of the VMAT model in the prediction of the IMRT plan was not demonstrated to be any worse than that predicted by the IMRT model. This might be because each of the VMAT plan comprised 178 control points in the training dataset, which allowed the VMAT model to capture more spatial information when utilized for plan optimization. The dose distribution predicted by the model for the IMRT plan was comparable to the dose distribution expected for an IMRT plan with 178 small fields. Therefore, the technique of using the VMAT model to predict IMRT plans does not impose any limitations on the dose distribution [13].

The two groups of IMRT automated plans demonstrated superior control of the low-dose region than the M-IMRT, as evident from results in Table 2 and Figure 4. Wu Hao et al. [13] provided an algorithmic explanation for this finding. The IMRT automated plans of the two groups produce comparable results. V-IMRT had slightly better control over some OARs than I-IMRT. The MU of the V-IMRT plan did not increase as a result of the optimization model, nor did the RV region V5% increase, but V-IMRT had greater control over the average dose compared to the I-IMRT, as evident from Table 3.

**Table 3:** Comparison of RV and MU between the IMRT automated plans in the two groups (*x*¯ ± *s*)

Item	V-IMRT	I-IMRT	*P* value
RV			
Dmean (Gy)	2.94 ± 1.51	3.07 ± 1.65	0.02
V5Gy (cc)	3974.44 ± 1114.31	3902 ± 1085.85	0.11
MU	860.4 ± 139.26	860.9 ± 163.97	0.98

The most significant advantage of KBP is its ability to reduce planning time [25]. During the optimization phase, manual plans necessitate several adjustments and iterative optimization. A prostate plan normally needs to be optimized 2-3 times, for about 2 hours, by a skilled physicist to satisfy clinical standards. In contrast, it typically takes 3-5 minutes to produce the plan using optimization based on the KBP model [26]. Planning takes less time, and the resulting plan is more effective as a result.

Despite the variety of treatment options available, radiation therapy including particle implantation and external radiation are still the main treatment methods for prostate cancer [17, 19]. In external radiation treatment, the professional backgrounds of radiotherapy practitioners are now diverse, and the clinical experience, literature knowledge, and understanding of clinical goals of practitioners all contribute to a high level of uncertainty in the quality of radiotherapy plans. The impact of individual subjectivity would be significantly reduced, and plan quality would increase through the implementation of objective and uniform evaluation procedures [27]. Automated planning can establish standards for plan development for junior radiotherapy planners, improve plan stability for senior planners, and lessen fluctuations in plan quality. In this study, the migration prediction ability of the model is confirmed, which is also conducive to improving the efficiency of the plan.

In fact, automated strategies make it easier to create standardized databases and collaborate across multiple centers. In addition to enhancing the homogeneity of treatment plans, it can serve as a benchmark for the evaluation and sharing of plans among facilities [28]. However, in this research, we only conducted a study of single-center cases and further verification is needed to see whether the same results can be obtained by model of cases from multiple centers or even from multiple countries. Further research is already underway.

## Conclusion

The KBP prediction model that was employed in the study is simply a DVH prediction model that was created through machine learning of the correlation between the DVH of the a priori plan and the position and volume of the target structure. The model can act on the optimization conditions to achieve automatic optimization of the plan. It increases the effectiveness of the plan production process while preventing fluctuations in the quality of the plan due to subjective factors involving the plan developers. This study’s primary focus was on model transfer training’s prediction outcomes. The development of the IMRT and VMAT prediction models were both successfully used to predict the IMRT plan. The study also included manual plans at the same time, and it was concluded that both the VMAT and IMRT automated plans of KBP for prostate had dosimetric advantages over manual plans. In comparison to the IMRT model, the KBP VMAT model’s prediction of IMRT plans exhibited no disadvantage in terms of dose control of the target region, OAR, and RV area. It is thus feasible to use VMAT model transfer training for IMRT planning in prostate radiation. The benefit of model transfer prediction is that it eliminates the need to reproduce the model for different treatment techniques, e.g., IMRT vs VMAT, decreases the time required for model building, and increases clinical effectiveness.

## Data Availability

Not applicable.

## Abbreviations

IMRT: Intensity-modulated radiotherapy
VMAT: Volumetric-modulated arc therapy
KBP: Knowledge-based planning
PTV: Planning target volume
MUs: Monitor units
OARs: Organs at risk
VOI: Volume of interesting
RV: All volumes outside of the PTV
TPS: Treatment planning system
GED: Geometry-based expected dose
DVH: Dose–volume histogram
CT: Computed tomography
BEV: Beam’s eye view
AAA: Anisotropic algorithm
GEDVH: Geometric DVH
CD: Cook’s distance
SR: Studentized Residual Values
MZ: Modified Z-score
CI: Conformal index
Lt/Rt: Left/right
PCS1: First primary component score
